# Delayed Interval Delivery of a Second Twin after the Preterm Labor of the First One in Twin Pregnancies: Delayed Delivery in Twin Pregnancies

**DOI:** 10.1155/2012/573824

**Published:** 2012-06-14

**Authors:** Yunus Aydin, Murat Celiloglu

**Affiliations:** ^1^Department of Obstetrics and Gynecology, Eskisehir Osmangazi University Medical Faculty, 26000 Eskisehir, Turkey; ^2^Department of Obstetrics and Gynecology, Dokuz Eylul University Medical Faculty, 35000 Izmir, Turkey

## Abstract

A diamnionic dichorionic twin pregnant women (due to in vitro fertilization) admitted to emergency department at the 21st week of gestation because of regular contractions. By gynecological examination, we observed 8 cm dilated cervix with 80% effacement. Amniotic membrane was also bulging through the cervix. After evaluation delivery of the presenting fetus occurred quickly. The baby's weight was 610 gr and no heart activity was detected. Placenta of the first fetus expulsed immediately. We decided to retain the second fetus to allow the improvement in the outcome. McDonald cerclage was performed and the patient treated with tocolytics and antibiotics, and she was continuously monitored up to the 28th week of pregnancy. After she was discharged in the 28th week, she was controlled weekly in obstetrics clinic. At the 36th gestational week which was 101 days after the cerclage procedure, 3639 g male fetus was delivered with cesarean section and had an uneventful neonatal course. Delayed-interval delivery is useful and acceptable therapeutic option for the management of the remaining fetus in twin pregnancies even after the expulsion of the placenta. Antibiotic and tocolytic administration with cervical cerclage application can be associated with longer interdelivery interval.

## 1. Introduction

Preterm delivery is associated with high risk for neonatal mortality and morbidity [1]. In twin pregnancies, there is a high risk of preterm delivery, that is, about 4%, 8%, 16% for delivery before the 30th, 32th, and 34th weeks, respectively, [2]. In the last decades, the number of multiple pregnancies have increased as a result of assisted reproductive technologies [3]. As a result, preterm labor in the second trimester and the premature rupture of membranes of the presenting fetus have now been encountered more commonly by perinatologist. Delivery of the presenting fetus in multiple gestation is usually followed by delivery of the second fetus or fetuses shortly thereafter [4]. Although case reports have demonstrated that delayed interval delivery can be successfully achieved in selected cases, optimal management is not clearly defined [4–11]. We report a case of delayed delivery with a interval of 101 days after the delivery of the first twin.

## 2. Case Report

A diamnionic dichorionic twin pregnant women (due to in vitro fertilization pregnancy) admitted to emergency department at the 21st week of gestation because of regular contractions. She had two embryo transfers after a successful ART cycle.

Pelvic examination revealed bulging membranes and a dilated cervix at 8 cm. Thereafter rapid delivery of the presenting fetus occurred. Delivered fetus weighed 610 g and no heart activity was detected. Placenta of the first fetus expulsed in 2-3 minutes after delivery easily. Patient was informed about the option for delaying the remaining fetus and also the complications of treatment for fetus and for herself.

Amniotic membrane of the remaining fetus was intact and the fetal heart monitorizations were normal during the followup. Plasental and cervical cultures were taken. Consequently vagina and the lower pole of the remaining sac were rinsed with iodized serum and a McDonald cerclage was performed under general anaesthesia. Bed rest, tocolysis with 25 mg indomethacin four times a day for two days, nifedipine 4 × 20 mg per day, and a 1.5 gr amoxicillin-clavulanic acid four times a day were administered for ten days while she was hospitalised. In addition, fetal lung maturity was induced by corticosteroids (12 mg betamethasone intramuscularly twice a day). Patient was continuously monitored through clinical assessment (blood pressure, heart rate, and temperature) and laboratory tests (blood cell count, C-reactive protein). Cervical length was monitored weekly with transperineal ultrasonography. Cervical cultures were also taken weekly. Daily fetal monitoring and weekly ultrasounds confirmed fetal growth and wellbeing. As she had no signs of infection according to the physical and lab findings, she was discharged at the 28th week of pregnancy. She was advised to take nifedipine 20 mg four times a day. Transperineal ultrasound revealed 27 mm cervical length at the time of discharge of the patient. After her discharge from the hospital we controlled the patient weekly with leukocyte count, C-reactive protein level, obstetric ultrasound, and also with transperineal ultrasound for cervical length. At each visit we also concentrated on if there were uterine contractions. Nifedipine treatment was stopped at the 34th week. At the 36th gestational week, which was 101 days after delivery of the first fetus, other fetus was delivered with cesarean section due to regular contractions and breech presentation. The second baby boy weighed 3639 g. Cervicalcerclage suture was also removed during cesarean section. Apgar score was 9 at first minute and he had an uneventful neonatal course.

## 3. Discussion

Gestational age is the most important predictor of neonatal survival in infants delivered before the 25th week of gestation. Prolongation of gestational period and increase in fetal weight significantly improve the fetal outcome [12, 13].

In recent years, an increasing number of delayed interval deliveries has been reported. In these reports, authors presented patients with similar conditions: multifetal gestation with delivery of the first fetus before the 30th week, diamniotic relationship between the presenting and subsequent fetus or fetuses, intact membranes in the remaining gestational sac, and also absence of fetal distress, lethal anomaly, abruptio placenta, intra-amniotic infection, or maternal indication for delivery [12–16]. Zhang et al. addressed to delayed interval delivery in twins in United States between 1995 and 1998 [14]. It was concluded that when a first twin was delivered at the 22th to 23th weeks, delayed delivery of the second twin was associated with reduced perinatal and infant mortality. Rosbergen et al. published a study of 24 cases of attempted delayed interval delivery. The mean of the delay was 19.9 days and the study showed a significant increase in birth weight and neonatal survival as well as decrease in adverse outcome [15]. Van der Straeten et al. reported that delayed delivery of the second fetus was associated with 13.4% mortality decrease in six cases [8]. Kalchbrenner et al. published a study of 7 cases and reported that the average birth weight, gestational age, and the duration of ventilatory support were significantly reduced with delayed delivery of the second twin with a mean of delivery interval of 36.2 days [4]. Fayad et al. reported a study of 35 cases with a mean interval of 47 days and the survival of the second twin reached to 78.6% and the mean birth weight of the second twin was 1217 g [6]. In one of the largest retrospective series Oyelese et al. reported findings about impacts of delayed interval delivery on perinatal mortality and morbidity in 4257 twins [16]. According to their findings, decreases in perinatal and infant mortality were observed only when the first twin was delivered at 22 to 23 weeks and when the delivery interval was ≤3 weeks. However, for intervals ≥4 weeks or when the first twin was delivered at 24 to 28 weeks (regardless of delivery interval), there was no benefit in perinatal or infant mortality. Delayed delivery of ≥4 weeks was associated with increased risk of small-for-gestational-age birth in the second twin, regardless of gestational age at delivery of the first. In our case second fetus delivered as 3639 g, so he was appropriate for gestational age. Long-term outcomes of infants who had been delivered as a consequence of delayed interval delivery were lacking. Silent intra-amniotic infection may cause periventricular leukomalacia, intraventricular hemorrhage, and cerebral palsy [17, 18]. Small-for-gestational-age may be a sign for long-term sequela that will appear in future. Long-term followup of these fetuses will probably clarify the risks.

Optimal management of delayed interval delivery is not defined yet. Cerclage, tocolysis, hospitalization, and antibiotic therapy are all controversial. Use of prophylactic cerclage in multifetal pregnancies has failed to show any benefitin some studies [19]. However, in a review of seven case series, Zhang et al. suggested that in cases of delayed interval delivery, immediate cervical cerclage after the first delivery was associated with a significantly longer interdelivery interval [12]. Studies in which cerclage was infrequently used reported a shorter interdelivery interval compared to studies where cerclage was used in all cases (the median was equal to 9 days versus 26 days, resp.). Cerclage may provide stability to the cervix; furthermore, it may minimize the exposure of fetal membranes to vaginal bacteria and acidity [11]. In these reviews, all studies included and used a broad-spectrum prophylactic antibiotics and parenteral tocolysis and found no evidence of an increased risk of intrauterine infection after cerclage. Fayad et al. observed that the mean interval delivery tended to be longer after high ligature of the cord, expulsion of the first twin's placenta, antibiotic therapy, and cerclage, although the differences were not statistically significant [6]. Arabin and van Eyck reported mean delay as 19 days (1–107) in 38 twin pregnancies by totally abstaining from cerclage in a prospective cohort study [20]. Concerns of chorioamnionitis due to closure of cervix may direct clinicians treatment plan, but in our patient we directly performed cervical cerclage. Still there is no consensus about performing cerclage in case of delaying fetus or fetuses in multifetal pregnancies. Randomized controlled trials will probably solve this conflict.

Our case was unusual in another point that, although no effort was applied but placenta of the first fetus expulsed spontaneously 2-3 minutes after delivery of the first fetus. In the literature it was mostly emphasized that the usual approach is high ligation of umbilical cord of the first fetus [20].

Delayed interval delivery is a useful and acceptable therapeutic option for the management of the remaining fetus in twin pregnancies even after the expulsion of first fetus's placenta. Antibiotic and tocolytic therapy with cervical cerclage application can be associated with longer interdelivery interval without increasing the risk of infection.
